# Smart Pack: Online Autonomous Object-Packing System Using RGB-D Sensor Data

**DOI:** 10.3390/s20164448

**Published:** 2020-08-09

**Authors:** Young-Dae Hong, Young-Joo Kim, Ki-Baek Lee

**Affiliations:** 1 Department of Electrical Engineering, Ajou University, Suwon 443-749, Korea; ydhong@ajou.ac.kr; 2 Korea Railroad Research Institute, Uiwang 437-757, Korea; osot@krri.re.kr; 3 Department of Electrical Engineering, Kwangwoon University, Seoul 01897, Korea

**Keywords:** object-packing, 3D bin packing problem, online optimization, RGB-D sensor

## Abstract

This paper proposes a novel online object-packing system which can measure the dimensions of every incoming object and calculate its desired position in a given container. Existing object-packing systems have the limitations of requiring the exact information of objects in advance or assuming them as boxes. Thus, this paper is mainly focused on the following two points: (1) Real-time calculation of the dimensions and orientation of an object; (2) Online optimization of the object’s position in a container. The dimensions and orientation of the object are obtained using an RGB-D sensor when the object is picked by a manipulator and moved over a certain position. The optimal position of the object is calculated by recognizing the container’s available space using another RGB-D sensor and minimizing the cost function that is formulated by the available space information and the optimization criteria inspired by the way people place things. The experimental results show that the proposed system successfully places the incoming various shaped objects in their proper positions.

## 1. Introduction

Object-packing is one of well-known bottlenecks in logistics automation. In recent years, most of the standard box-based logistics transportation has been successfully automated based on specially designed warehouse and mobile robot platforms. However, packaging individual products in boxes still requires labor. The packaging process is divided into two tasks: object-picking and packing. The former means picking one of the items randomly arranged within a container, and it has been tried to be automated in various directions with deep learning including the recent Amazon robotic challenge outcomes [1,2,3]. The latter implies placing the picked up item in the most suitable position in another container, and mostly it has been implemented in a theoretical way or in a hybrid form with human collaboration [4,5].

Accordingly, it is necessary to focus on developing an autonomous object-packing mechanism, assuming a well-implemented object-picking system (hereafter referred to as the picker) in the previous step. The objects must be handed over one by one from the picker and placed at their proper positions in a box of fixed size. This task can be classified as a bin packing problem (BPP), which is a classical combinatorial optimization problem. The BPP is known to be NP-Hard and has been consistently studied since the 1970s [6,7,8]. In addition, this task could be termed as 3D BPP depending on the dimensions being used for maximizing the space use [9]. Moreover, this task is an online BPP because the dimensions and orientations of objects are unknown before they are picked by the picker [10,11].

**Figure 1:**
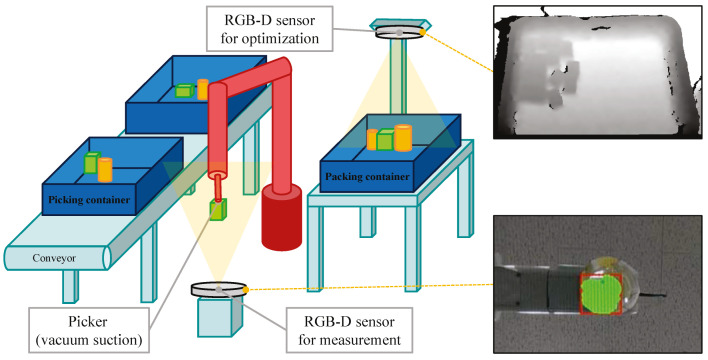
The illustration of the proposed system and the examples of the data from the sensors.

Thus, in this paper, we propose an online autonomous object-packing system using RGB-D sensor data. The main contributions of this work are two-fold. One is the method for real-time measurement of objects. Figure 1 illustrates the configuration of the RGB-D sensor for the measurement and an example of the data from the sensor. When the picker moves to the center of the sensor frame, a depth frame is captured from the sensor. After extracting the object area from this depth frame, the 2D contour and the depth of the object are calculated using the point cloud set of the object area. The other is the online optimization algorithm for the placement of each picked object. Figure 1 also demonstrates the configuration of the RGB-D sensor for the available container space recognition and example of the data from the sensor. The proposed algorithm is inspired by the way people load things. It is impossible to obtain a global optimum without information on all objects. If the global optimum cannot be obtained, it is best to find an efficient local optimum that minimizes the computation. In this situation, a person tries to place things in the lowest space possible and bring them together. The proposed algorithm is designed by reflecting these human behaviors in optimization criteria.

The rest of the paper is organized as follows. Section 2 reviews the related works related to online as well as offline 3D BPP. Section 3 discusses the method for real-time measurement of objects, and Section 4 details the online optimization algorithm for determining the position of each picked object. Section 5 provides the experimental results on both the real-time object measurement and the online object placement optimization. It presents the statistical analyses about the accuracy and computation time of the measurement. In addition, the effectiveness of the online optimization is demonstrated with respect to the scenario of the sequential inputs of randomly generated virtual objects. Section 6 concludes the work.

## 2. Related Works

A huge number of methods exists in the literature for packing problems. Most of these are offline methods. Offline means the arrangement of objects are optimized at once based on the previously obtained dimension information of them. In the previous offline methods, the position of every object has been formulated as a multi-dimensional solution vector. Then, along with the dimension information, the solution has been figured out through optimization algorithms such as deep reinforcement learning, Quasi-Monte-Carlo tree search, genetic algorithm, space-defragmentation heuristic approach, or beam search algorithm [9,12,13,14,15]. Since they have assumed objects as boxes, the orientations of the objects have not been considered important. These offline methods are logically straight forward and guarantee the precise solution. On the other hand, if all the information about objects has not been obtained in advance, they cannot be used. Thus, we have taken advantage of only optimization concept from these offline methods since the object need to be passed and placed one by one for fully automated object packing.

Online BPP has been also studied extensively in the literature. The previous studies successfully implemented online packing processes. Some have employed the fine-tuned heuristics based on the product packing routine [16,17,18] and the others have actively used deep reinforcement learning for their methods to learn how to pack things effectively themselves through trials and errors [11,19,20,21]. However, due to the high computational load of online optimization or learning complexity, objects have been assumed to be boxes without rotations or 3D problem has been decomposed into multiple 2D problems by packing objects layer by layer. Also, the container has not been monitored since the packing process has been assumed to be ideal. In this paper, considering these issues, each object dimension (including orientation) as well as the corresponding available packing area of a container is measured in real time as a preliminary step of online object position optimization. In addition, the optimization problem has been simplified using the optimization criteria inspired by the way people load things.

## 3. Real-Time Object Measurement

In online 3D BPP, object information can be obtained after the picker delivers the object. For simplicity, it is assumed that a well-implemented object-picking system is virtually implemented. As the picking mechanism, one-point vacuum suction is assumed. RGB-D sensors are effective for quickly and inexpensively measuring the dimensions and orientation of an object, as demonstrated by previous studies [22,23,24]. In this section, we first describe the configuration of the picker and an RGB-D sensor. In addition, the procedure for obtaining the dimensions and orientation of an object from sensor data is explained.

### 3.1. The Configuration of the Picker and the RGB-D Sensor

**Figure 2:**
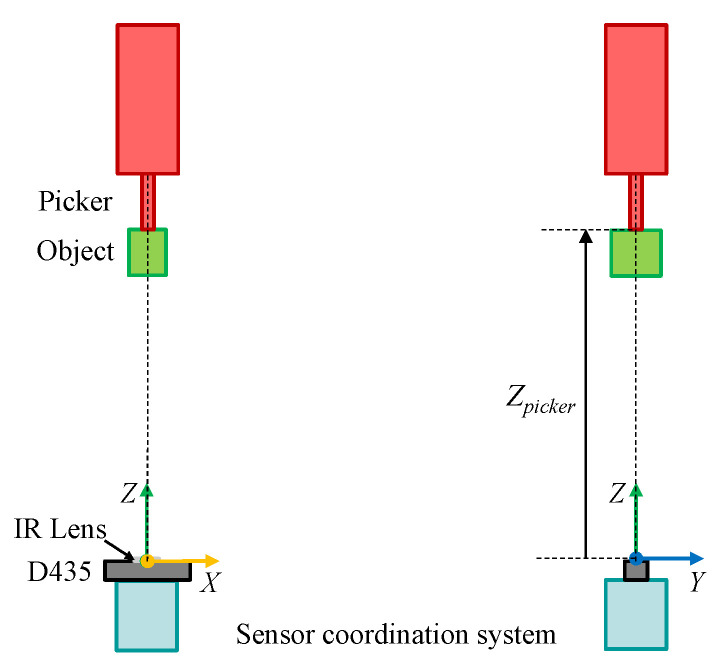
The configuration of the picker and the RGB-D sensor.

Since the picker picks an object from the top, it is natural that the RGB-D sensor for measurement is installed at the bottom of the object as illustrated in Figure 1. As the RGB-D sensor, Intel RealSense D435 has been employed. Among the specifications of the D435, the important factors for this configuration are depth field of view and minimum depth distance. The depth field of view is 86°×57°(±3°) and the minimum depth distance is 0.105 m. In consideration of these, the centers of the picker and the D435 infrared lens in xy-axes should match, and the distance $Z_{picker}$ between the lens and the lowest position of the picker in *z*-axis should be large enough. In this paper, the maximum depth of objects $D_{obj}$ is assumed to be 0.3 m and $Z_{picker}$ is set to 1 m where the origin of xyz-axes is on the top center of the lens. Figure 2 shows the configuration of the picker and the RGB-D sensor in detail.

### 3.2. The Object Measurement Procedure

The overall procedure of the object measurement is summarized in Algorithm 1, and each step of the algorithm is explained in the following.
**Algorithm 1** Object Measurement $Z_{picker}$: The lowest position of the picker from the lens in *z*-axis $D_{obj}$: The maximum depth of objects rows, cols: The rows and columns in the 2D pixel coordinate system d(rows,cols): The depth value at (rows,cols) $A_{min}$: The minimum area of object cross section *C*: The contour of the object w,h,d,ϕ: The width, height, depth and orientation of the object W,H: The width and height of the depth frame $\left(p_{x},p_{y}\right)$: The picked position in xy-axes on the object  (1) Acquire sensor data. Capture a depth frame from D435 **if** the depth frame is not successfully captured **then**     return the error code of ’−1’ **end if** Calculate point clouds from the depth frame  (2) Extract object area. Separate the pixels where $Z_{picker}-D_{obj}<d\left(r,c\right)<Z_{picker}$ Find the contours of the separated pixels whose area is larger than $A_{min}$ **if** no contour is found **then**     return the error code of ’−2’ **end if** Select the largest contour as *C* **if**
*C* is out of the depth frame bound **then**     return the error code of ’−3’ **end if**  (3) Calculate *d*, *w*, *h* and ϕ. Accumulate the point clouds in *C* $z_{avg}$ = average of the *z*-axis values of the point clouds *d* = $Z_{picker}-z_{avg}$ Find a rotated rectangle of the minimum area enclosing *C* Calculate the scaling factor *S* from the point clouds *w* = width pixels of the rectangle ×S *h* = height pixels of the rectangle ×S ϕ = rotation of the rectangle  (4) Calculate $p_{x}$ and $p_{y}$. $\left(c_{x},c_{y}\right)$ = average of the four corner positions of the rectangle in pixels $\left(d_{x},d_{y}\right)$ = ($c_{x}$ − W/2, H/2 − $c_{y}$) $\left[\begin{array}{c} p_{x}\\ p_{y} \end{array}\right]=-S\left[\begin{array}{l} cos(\phi )\;-sin(\phi )\\ sin(\phi )\;\;\;\;\;cos(\phi ) \end{array}\right]\left[\begin{array}{c} d_{x}\\ d_{y} \end{array}\right]$  (5) Return *w*, *h*, *d*, ϕ, $p_{x}$ and $p_{y}$

#### 3.2.1. Acquire Sensor Data

First, a depth frame is captured from D435. If the captured frame is not normal, the error code of ‘−1’ is returned. Then, from the depth frame, the corresponding point clouds are calculated.

#### 3.2.2. Extract Object Area

**Figure 3:**
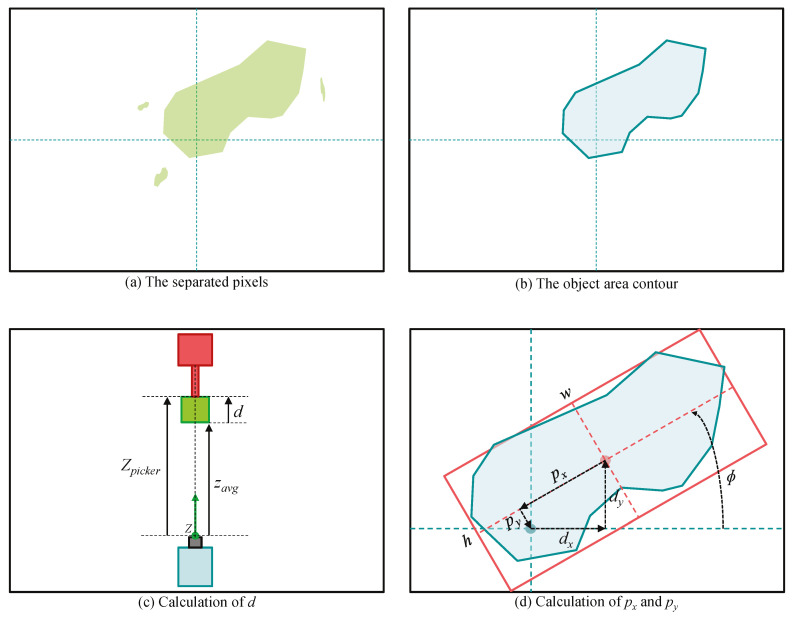
The procedure of the object measurement.

From all the pixels in the depth frame, the pixels which are in the object area are separated. As  shown in Figure 2, the depth values inside the object area lies between $Z_{picker}-D_{obj}$ and $Z_{picker}$. After that, the contours enclosing the separated pixels are obtained. Among these, the contours with the area size of less than $A_{min}$ are filtered out considering sensor noises. Since the object is carried one by one, the largest contour should represent the object area. If no contour is detected, the error code of ‘−2’ is returned. In addition, if the object area is out of the depth frame bound, the error code of ‘−3’ is returned. The examples of the separated pixels and the contour of the object area are described in Figure 3a,b, respectively.

#### 3.2.3. Calculate *d*, *w*, *h*, and ϕ

As shown in Figure 3c,d can be calculated by subtracting $z_{avg}$ from $Z_{picker}$. $z_{avg}$ is the average of the *z*-axis values of the point clouds in the object area. And then, a rotated rectangle of the minimum area enclosing the object area is obtained. By scaling the dimensions of this rectangle from the pixel unit to real world unit, *w*, *h*, and ϕ can also be calculated.

#### 3.2.4. Calculate $p_{x}$ and $p_{y}$

Since the picker cannot always pick the object at the center of the object, the picked position in xy-axes on the object ($p_{x},p_{y}$) should also be calculated. They can be carried out by a translation and a rotation as shown in Figure 3d.

#### 3.2.5. Return $w,h,d,\phi ,p_{x}$ and $p_{y}$

Finally, $w,h,d,\phi ,p_{x}$ and $p_{y}$ are returned.

## 4. Online Object Placement Optimization

In online 3D BPP, the optimal position of the incoming object should be calculated as soon as possible. Since there is no information about the remaining objects that have not been picked up, it is impossible to perform global optimization using only incoming and packed object information. At this point, it may be better to simplify the problem as much as possible. A guidance for this simplification can be inspired by the way people place things in a container. In general, people try to: (1) place things in the lowest space possible and (2) bring them together. These human behaviors are reflected in the proposed optimization process. In this section, the optimization criteria and the corresponding cost function considering the human behavior are explained. Then, the overall process of the optimization algorithm is described step by step.

### 4.1. The Optimization Criteria

**Criterion 1**: Place things at the lowest position   

**Figure 4:**
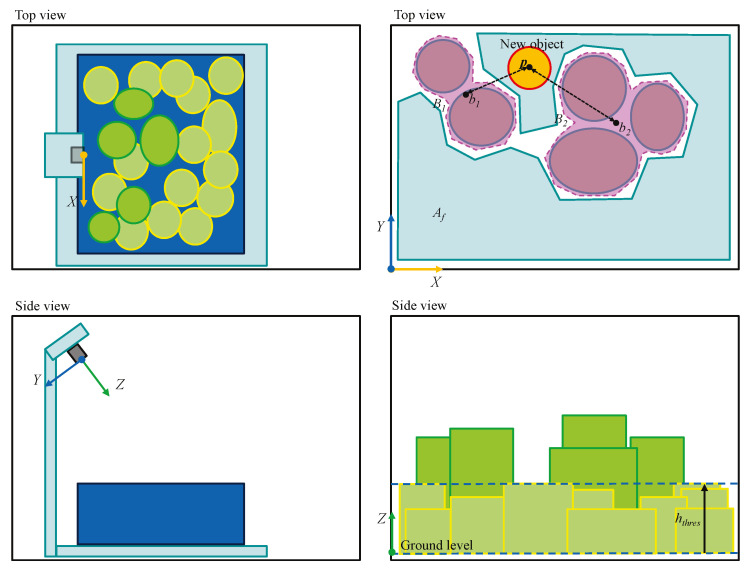
The illustration of the optimization criteria.

Let $h_{thres}$ be a threshold height value and $A_{f}$ be the current feasible packing area in container(global) coordinate system. The feasible packing area means a set of the 2D positions at which the object with contour *C* can be placed free of side contact with others if it is above $h_{thres}$ height. An example of $A_{f}$ is described in Figure 4. To place things at the lowest position, we need to find the position vector $p\in A_{f}$ with the smallest $h_{thres}$.

   **Criterion 2**: Bring things together   

Let $B_{i}$ be the *i*-th segment enclosing of adjacent objects as shown in Figure 4. We can make the objects together by minimizing $\sum dist(p,b_{i})$ where dist(j,k) means the distance between *j* and *k* and $b_{i}$ is the center of mass of $B_{i}$.

   **Cost Function**   

By combining Criteria 1 and 2, the cost function can be derived as follows:

(1) $$cost\left(p,h_{thres}\right)=\left\{\begin{array}{cc} K_{1}\cdot h_{thres}+K_{2}\cdot \sum dist\left(p,b_{i}\right) & \mathrm{if}\;A_{f}\neq \emptyset \\ 1,000,000 & \mathrm{if}\;A_{f}=\emptyset \end{array}\right.$$

where $K_{1}$ and $K_{2}$ are the scaling constants. We set this problem as a minimization problem and this cost function is a function to be minimized. At first, $A_{f}$ should be checked. If $h_{thres}$ is too small, as described in right side sub-figures of Figure 4, $A_{f}$ becomes empty set which means that there is no feasible packing area with that $h_{thres}$ value. In this case, the corresponding cost function value is 1,000,000 which is an exceptionally high value meaning failure. In contrast, if $A_{f}$ is not an empty set, the cost function value is calculated by a linear combination of $h_{thres}$ and $\sum dist(p,b_{i})$. Based on this equation, the smaller the both $h_{t}hres$ and $\sum dist(p,b_{i})$, the better. This is logically consistent with the criteria. In this paper, $K_{1}$ and $K_{2}$ are respectively set as 1000 and 1.

### 4.2. The Overall Process of the Optimization Algorithm

The overall process of the optimization is summarized in Algorithm 2, and each step of the algorithm is explained in the following.

#### 4.2.1. Acquire Sensor Data

First, a depth frame is captured from D435. If the captured frame is not normal, the error code of ’−1’ is returned. Then, from the depth frame, the corresponding point clouds are calculated.

#### 4.2.2. Generate 2D Depth Map

First, the 3D point clouds are transformed from the sensor coordinate system (SCS) to the container(global) coordinate system (CCS). SCS and CCS are respectively described in the left and right sub-figures of Figure 4. Then every point cloud ($x_{i},y_{i},z_{i}$) is projected into 2D depth map by setting the value at ($\left\lfloor x_{i}\right\rfloor$, $\left\lfloor y_{i}\right\rfloor$) to $z_{i}$. Since the point clouds cannot always cover every pixel in 2D depth map, the empty pixels are sequentially occupied by linear interpolation with the adjacent pixels. In addition, 3×3 sized mean filter is applied to the map image to reduce noises. At last, $A_{f},B_{i}$ and $b_{i}$ are obtained according to p and $h_{thres}$ as described in the right sub-figures of Figure 4 based on this 2D depth map and the measured constants $w,h,d,\phi ,p_{x}$ and $p_{y}$.

#### 4.2.3. Run Differential Evolution (DE) Algorithm

In this paper, as the optimization algorithm, differential evolution (DE) is employed. DE is a well-known optimization algorithm for its effectiveness in multi-dimensional single-objective optimization problems [25,26,27]. By the benefit of the optimization criteria inspired by human behavior, the object placement optimization problem becomes simplified into a low-dimensional single-objective optimization problem and be able to solved by DE. At first, the DE algorithm is initialized with appropriate configuration parameters. Then, for each solution candidate, the processes (1) and (2) are performed and the cost function is calculated. Each solution candidate is formulated as a vector of $\left(h_{thres},p\right)$ and becomes the input of the cost function. The cost function output is the better the smaller. After that, the solution candidates are updated by DE update process. This update process is repeated until the termination condition is met. As a result, the final solution along with its cost function value is stored.
**Algorithm 2** Optimization algorithm SCS: The sensor coordinate system CCS: The container(global) coordinate system  (1) Acquire sensor data. Capture a depth frame from D435 **if** the depth frame is not successfully captured **then**     return the error code of ’−1’ **end if** Calculate point clouds from the depth frame  (2) Generate 2D depth map. Transform the 3D point clouds from SCS to CCS Project every point cloud ($x_{i},y_{i},z_{i}$) into 2D depth map by setting the value at ($\left\lfloor x_{i}\right\rfloor$, $\left\lfloor y_{i}\right\rfloor$) to $z_{i}$ Occupy the empty pixels of the map image by linear interpolation with the adjacent pixels Apply 3×3 sized mean filter to reduce noises  (3) Run differential evolution (DE) algorithm. Initialize the DE algorithm Set input bounds of $h_{thres}$ and p Select appropriate configuration parameters **while** the termination condition is not met **do**     **for** each solution candidate **do**         Perform (1) and (2)         Obtain $A_{f}$ with respect to $h_{thres}$ using the generated depth image         Calculate the cost function     **end for**     Update solution candidates using DE update process **end while** Store the final solution along with its cost function value  (4) Return $h_{thres}$ and p. Repeat (3) three times Return the solution ($h_{thres}$, p) with the smallest cost function value

#### 4.2.4. Return the Optimal $h_{thres}$ and p

Unfortunately, DE cannot always guarantee the solution convergence. To complement this issue, in this paper, the DE algorithm is run three times in a row and the solution set with the smallest cost function value is selected. Since the problem is simplified enough as mentioned above, the computational load for the three runs keeps affordable.

## 5. Experiments

In the experiments, the proposed system was implemented as a software written in Python (version 3.5.2) language with Numpy and Scipy libraries. The software was run on Linux OS (version 16.04) with Intel i7-6900K CPU, 128GB DDR4 RAM, and NVIDIA Titan X Pascal GPU. In this section, the detailed information about the environment settings and then the experimental results are provided. Please note that since the object sequence and computational environment used by the existing methodologies has not been disclosed, the comparison by quantitative metrics has been practically limited. However, the main advantages compared to the existing methods from a qualitative viewpoint can be found as the following three. First, a novel method for measuring the dimensions of objects in real time has been proposed. In the existing methods, it was assumed that the measurement was completed in advance. However, before this paper, there have not been any studies for measuring an object in real time during the process from picking to packing. Second, rotation is now possible when the object is placed. The container for packing as well as the objects is not a sharp cuboid and the sizes of the objects also vary. In addition, the actuation errors of the manipulation robots cannot be ignored. In this condition, the effectiveness of rotating the object increases. Finally, it works robustly against various errors that are inevitable in reality. Since the container space is monitored in real time, the proposed method can reflect unexpected errors in optimizing the position of the next objects even if the robot moves the object slightly out of the desired position or the objects inside the container are tilted.

### 5.1. Real-Time Object Measurement

The proposed real-time object measurement system was tested with 6 kinds of beverages and they were repeatedly measured 20 times each. Due to the virtual picker, the objects were measured hanging from a steel frame. The measurement system had no prior knowledge of the objects at all. Since the previous studies did not consider the situation that the objects needed to be measured in real time during transferred by the picker, empirical comparison could not be done. Instead, the computation time and measurement accuracy are statistically analyzed.

As shown in Table 1, the worst computation time was 35.4 ms and the frames per second of about 30 was maintained. The average errors of width *w* and height *h* were relatively greater than the others. The depth image resolution of D435 is 1280 by 720, and considering the captured region of interest (ROI), the ideal accuracies for *w* and *h* are between 1.0 mm to 1.5 mm. In addition, infrared diffuse reflection at the edge of the object is not negligible, and hot noise, although not frequent, occurs. On the other hand, depth *d*, orientation ϕ, picking position $p_{x}$ and $p_{y}$ showed less errors by the averaging effect. In particular, the ideal depth accuracy of the D435 is around 1% of the distance from the object and if the sensor is installed 1 m from the object, the expected accuracy is between 2.5 mm to 5 mm. This implies that the proposed measurement system maximized the performance of D435. Meanwhile, since the picking process is assumed to be in ideal condition in this paper, this *d* error can cause a problem only if it is measured lower than the ground truth and the end effector is pressed from the bottom. However, vacuum suction pickers usually have an elastic spring damper in the joint, which can compensate for the displacement and the accompanying pressure due to an error within around 10 mm. Please note that the worst error in *d* was 4.8 mm. In addition, if the robot manipulator is controlled through the force and torque feedbacks, the allowable height error may be greater. As a result, the proposed system successfully measured the dimensions of the object in real time with sufficiently low error rate for practical uses.

### 5.2. Online Object Placement Optimization

The proposed online object placement optimization was tested using the refrigerated goods which were actually stored in GS Retail Corp. logistic center, South Korea, during December 2019. Due to the virtual picker issue, the test objects were also virtually generated by referencing the test products. Each test object was generated by inheriting the dimensions of a randomly chosen test product along with a random set of $p_{x},p_{y}$ and ϕ. The test objects were packed until $h_{thres}\geq H_{max}$ where $H_{max}$ is the maximum height of the container. Total 10 containers were packed for the test. Please note that $h_{thres}$ variable was quantized with amount of 10 mm and the parameters for the DE was set as the default settings in [27].

As shown in Table 2, the worst computation time was 346.7 ms. For example, if we employ Denso VS-650 robot as the picker, since it requires about 150.0 ms to move an object 1 m and return according to its specification, the proposed method can pack nearly two objects in one second. This implies that the proposed method is fast enough for online packing. In addition, the average container occupancy ratio was 63.2%. Since various refrigerated products stored in the actual warehouse were considered, this ratio can never be regarded as small.

**Figure 5:**
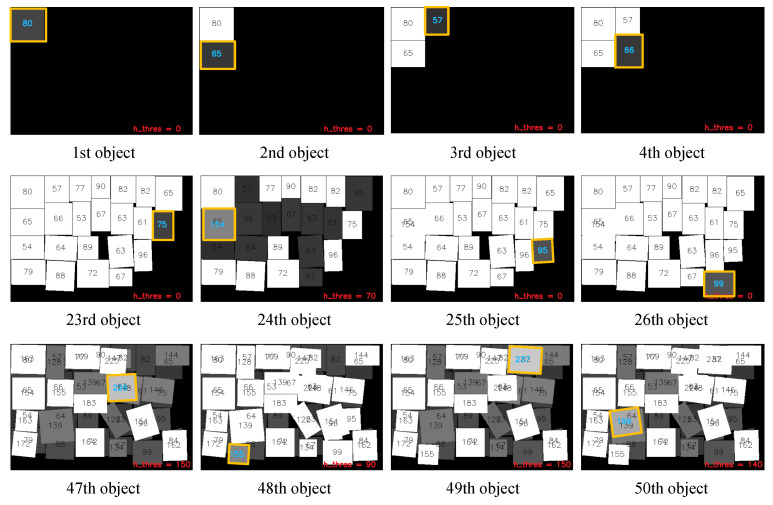
The snap shots of the object-packing simulation without manipulation error (object boundary margin of 1 mm).

Figure 5 shows the snap shots of the object-packing simulation without manipulation error. The corresponding object boundary margin was set as 1 mm. As shown in the figure, the rotation of the objects can be taken into account. The most important thing is that the proposed packing algorithm can make full use of multiple layers instead of the layer by layer strategies of the previous studies. The 24th object was placed on the second layer because the object was relatively large and the feasible area on the first layer was tight. Then, the 25th and 26th objects were placed on the remaining area of the first layer. Similar phenomena can be seen through the 47th–50th objects. As a result, the proposed method successfully performed online object-packing using a novel optimization process mimicking human behavior.

**Figure 6:**
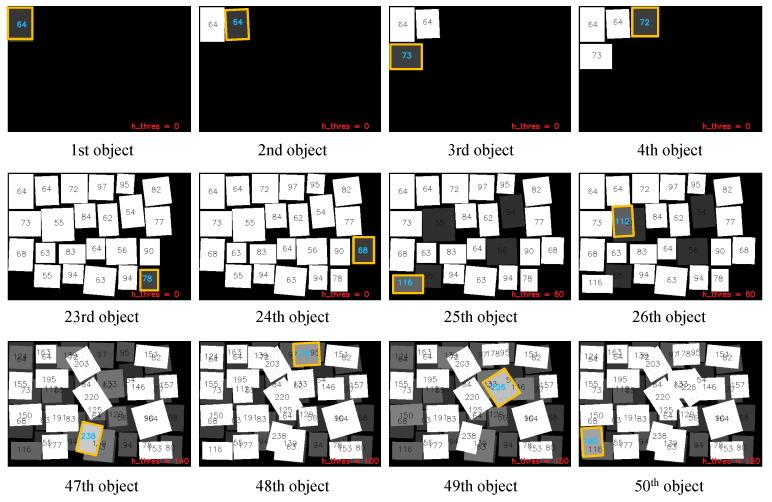
The snap shots of the object-packing simulation with manipulation error (position error of [−3, 3] mm, orientation error of [−3, 3]° and object boundary margin of 5 mm).

Figure 6 shows the snap shots of the object-packing simulation without manipulation error. The error was randomly generated (−3, 3) mm in position and (−3, 3)° in orientation. To take this error into account, the corresponding object boundary margin was set as 5 mm. As shown in the figure, the proposed method showed robust performance against the unexpected manipulation errors. This also implies that even though the object may be tilted when the robot manipulator releases the object, the proposed method monitors the container space in real time and is able to reflect it to the following object placement.

**Figure 7:**
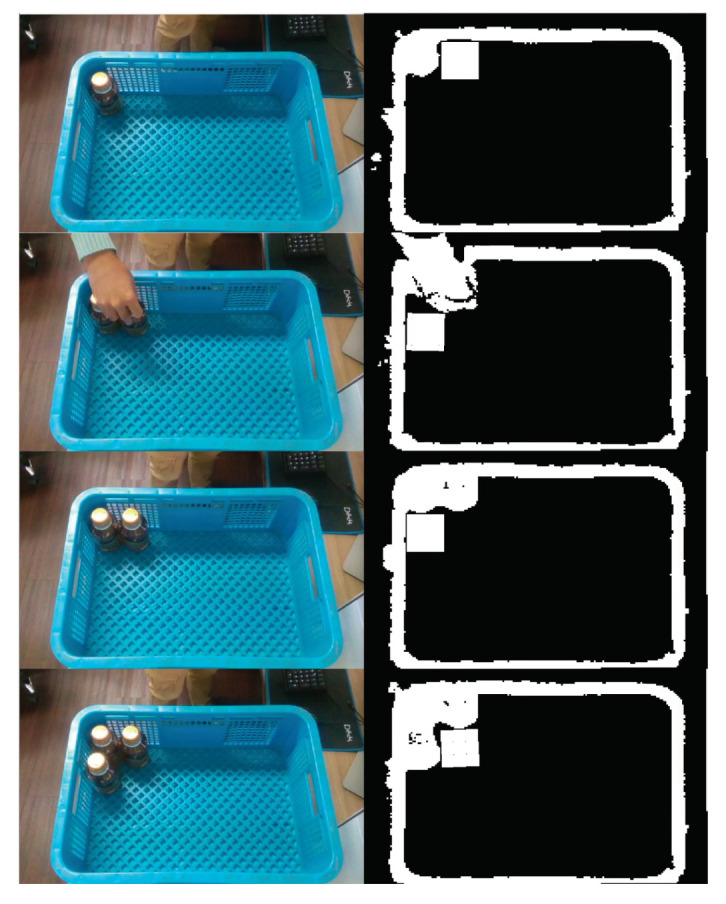
The snap shots of the actual object-packing process.

Finally, the actual object-packing process using a standard container and the products used in actual logistic is demonstrated in Figure 7. Left column shows the RGB images and the Right column shows the generated depth maps. The white square represents the placement position of the next object which is calculated through the proposed optimization method. Instead of a manipulator, the objects were moved by hand to the desired position. As shown in the figure, the proposed method successfully calculated the optimized placement position with respect to serial inputs.

## 6. Conclusions

In this paper, a novel online object-packing system was proposed. In the proposed method, the dimensions of every incoming object could be measured in real time and the desired position of the object could be optimized in online way. The dimensions and orientation of the object were carried out using an RGB-D sensor when the object was picked by a manipulator and moved over a certain position. The optimal position of the object was calculated by recognizing the container’s available space using another RGB-D sensor and minimizing the cost function that is formulated by the available space information and the optimization criteria inspired by human behavior. The experimental results showed that through the proposed method, the dimensions of the object were successfully measured in real time with sufficiently low error rate for practical uses. In addition, the objects were effectively packed at their desired position making full use of multiple layers. Most importantly, the proposed method had high practical potential in that various refrigerated products stored in actual warehouses were considered.

## Figures and Tables

**Table 1:** The computation time and error of the proposed system.

	Computation Time (ms)	Error					
		w (mm)	h (mm)	d (mm)	ϕ (deg)	$p_{x}$ (mm)	$p_{y}$ (mm)
Min	20.6	2.6	1.8	0.1	0.0	0.6	0.7
Max	35.4	7.6	7.7	4.8	5.9	7.5	7.4
Average	27.6	5.0	5.1	2.4	3.2	4.3	4.1
STD	4.4	1.4	1.6	1.4	1.9	2.3	2.1

**Table 2:** The computation time and container occupancy ratio of the proposed system.

	Computation	Container Occupancy
	Time (ms)	Ratio (%)
Min	227.1	52.2
Max	346.7	79.2
Average	293.5	63.2
STD	33.1	9.3
